# Patient Delay in Hospital Visiting and the Weekend Effect of Surveillance Report on Hand-Foot-and-Mouth Disease and Epidemic Parotitis in Hanzhong City, China

**DOI:** 10.1155/2020/7081219

**Published:** 2020-05-06

**Authors:** Jianjun Wei, Zhonghai Zhu, Qi Qi, Lingxia Zeng

**Affiliations:** ^1^Center for Disease Control and Prevention, Hanzhong, Shaanxi, China; ^2^School of Public Health, Xi'an Jiaotong University Health Science Center, Shaanxi 710061, China

## Abstract

**Background:**

We aimed at investigating the prevalence and associated factors of patient delay in hospital visiting and weekend effect of disease surveillance on hand-foot-and-mouth disease and epidemic parotitis/mumps.

**Methods:**

Daily report data on hand-foot-and-mouth disease and epidemic parotitis cases between January 1, 2014, and December 31, 2017, in Hanzhong, Shaanxi, China, were collected. The patient delay in hospital visiting was defined by the date difference between disease onset and patient's visit to hospital. Differences of delayed durations and percentages were compared by using nonparametric or *χ*^2^ tests across gender, age, occupation, disease classification, epidemic and nonepidemic seasons, and years of disease onset. Additionally, to determine whether there existed a weekend effect of disease surveillance, the mean cases reported on weekdays and weekends were also compared.

**Results:**

A total of 14,814 patients with hand-foot-and-mouth disease and 4013 with epidemic parotitis were recorded, respectively. We found that 43.1% of the hand-foot-and-mouth disease and 36.5% of the epidemic parotitis patients had delayed visiting to hospital. All patients were reported through the online surveillance system on the day of visiting hospital. The percentage of delayed visiting to hospital differed significantly by years and epidemic and nonepidemic seasons and between children in and not in childcare center (all *p* values <0.05). In addition, the reported numbers of both diseases fluctuated on weekdays but obviously decreased on weekends regardless of the epidemic or nonepidemic seasons.

**Conclusions:**

The reported cases of HFMD and epidemic parotitis had an obvious weekend effect, with an increasing tendency of cases delaying in hospital visiting over the recent years in Hanzhong, China. Parents and caregivers rather than health systems should be primarily targeted for the prevention and control of infectious diseases and their local outbreaks such as community-based education on the second-dose vaccination of mumps and/or hand hygiene.

## 1. Background

Epidemic parotitis/mumps is an acute infectious disease of the respiratory tract and characterized by a highly recessive infection rate and long infectious durations [1]. Patients in the early stage or recessively infected individuals are not easily identified resulting in a source of infection and may easily cause outbreaks or epidemic in collective units, such as schools and childcare centers [2]. China has included it in the disease surveillance information report management system since 2004 [3]. The reported incidence of epidemic parotitis between 2004 and 2010 in China fluctuated around 20/100,000 [4]. However, in 2008 and 2009, epidemic parotitis outbreaks accounted for approximately 21% of all outburst epidemics of infectious diseases in China, which increased to 34% in 2010 and 2012 [5–7]. In addition, students and children are the targeted populations of epidemic parotitis [3, 5, 6], accounting for approximately 93% of all cases each year. Epidemic parotitis outbreaks in schools took up approximately 99% of all epidemic parotitis outbreaks. Since 2004, the incidence of epidemic parotitis in China has been highest among children <15 years, which accounted for approximately 85% to 92% of all cases [3, 5, 6]. Among these children cases, the incidence was highest in children between 5 and 9 years, accounting for approximately 43% to 49% of all cases [8]. Key factors causing the constant epidemic include an insensitive surveillance system, improper implementation of relevant policies, and an inability to provide a timely diagnosis and reporting and effective quarantine for patients at the beginning of the disease onset [4].

Recently, the hand-foot-and-mouth disease (HFMD) epidemic in China has shown characteristics of a fairly high incidence rate and long peak durations and wide distribution of the epidemic [9]. After 2008, medical institutes in each level in China were required to report the HFMD cases directly through the online disease surveillance information report management system according to the “Law of the People's Republic of China on the prevention and treatment of infectious disease.” Cases with HFMD from children under 5 years of age constituted more than 80% of all cases with the highest incidence among children between 1 and 3 years [10, 11]. The most effective strategy to prevent the spread of HFMD is to strengthen surveillance reporting system of the disease epidemic with a focus on active surveillance of children under 5 years of age and ensure daily monitoring and reporting, in which patients would be identified, diagnosed, reported, quarantined, and treated as early as possible [12]. However, analyses on surveillance data from multiple locations showed a significant weekend effect on HFMD reporting [13, 14].

Because the two infectious diseases discussed above mainly target on infants and school-aged children, the surveillance quality, timeliness, and completeness of routine surveillance have great implications for follow-up managements of cases and health departments to prevent the incidence and even outbreaks of these infectious diseases. Along with the improvement of the direct online reporting system, the time difference between the disease diagnosis and recording formation in the online report system, i.e., diagnosis to report, usually is within one day in China. However, the time difference between disease onset and diagnosis at hospitals, i.e., onset to diagnosis, is an emerging issue that compromised the timely report of infectious diseases on the online reporting system [15, 16]. Given that these two infectious diseases easily developed into outbreaks, we hypothesized the time difference between disease onset and diagnosis and the potential weekend effect of epidemic reporting are probably the primary issues that affect the quality of infectious disease surveillance.

Based on daily report data for HFMD and epidemic parotitis cases between January 1, 2014, and December 31, 2017, in Hanzhong, Shaanxi, China, we aimed at investigating the prevalence and influencing factors of patient delay in hospital visiting and the possibility of a weekend effect of disease surveillance. We also aimed at comparing the durations of delay in hospital visiting for these two diseases between epidemic and nonepidemic seasons, which would provide evidence for the improvement of the surveillance quality and the control of the spread of these two diseases among young children.

## 2. Methods

### 2.1. Data Source

Data on HFMD and epidemic parotitis were obtained from the “Infectious disease report information management system” subsystem of the “China disease prevention and control information system” between January 1, 2014, and December 31, 2017. According to the regulations stated in the “Law of the People's Republic of China on the prevention and treatment of infectious disease” and “Standard for the management of infectious disease information report,” the first physician of receiving patients at every medical institution should fill out the online “Infectious disease report card of the People's Republic of China” once the patient was diagnosed with an infectious disease. The following information was collected including the patients' gender, date of birth/age, race, occupation, residential address, classification of diseases (clinically diagnosed cases and confirmed cases), and date of disease onset, diagnosis, and online report.

### 2.2. Diagnostic Criteria of HFMD and Epidemic Parotitis

HFMD was defined by the “Diagnosis and treatment guidelines of HFMD (2010 edition)” that was published by the Ministry of Health on April 27, 2010 [17]. Clinically diagnosed cases were patients who had disease onset during the epidemic season and usually were preschool children and infants. The common symptoms included fever and rash on the hands, feet, mouth, and hip. Clinically diagnosed cases were further confirmed as confirmed cases with the presence of any one of the following criteria: (1) a positive nucleic acid test of enterovirus, e.g., coxsackievirus A 16 (CoxA16) and enterovirus-71 (EV71), (2) isolated enteroviruses that were identified to be CoxA16, EV71, or other enteroviruses that might cause HFMD, and (3) the serum antibody titers of CoxA16, EV71, or other enteroviruses that might cause HFMD increased more than 4-fold during the acute and recovery phases.

The diagnostic criteria for epidemic parotitis were based on the “Epidemic parotitis diagnostic criteria, WS270-2007” which was approved by the Ministry of Health, People's Republic of China, and issued on October 15, 2007 [18]. Clinically diagnosed cases were defined using the following criteria: (1) swelling, pain, and/or amplifying pain of the unilateral, bilateral parotid glands or other saliva glands during mouth opening, chewing, and eating acidic food, (2) symptoms like fever, headache, fatigue, and loss of appetite, (3) contacting with individuals infected by epidemic parotitis 14 to 28 days before disease onset, and/or those living in a region with an ongoing epidemic parotitis and having symptoms like fever, headache, fatigue, and loss of appetite, and (4) elevated serum and urine amylase levels and/or changes of cerebral spinal fluid indicating the presence of viral meningitis. Laboratory-confirmed cases were further identified from clinically confirmed cases by the following criteria: (1) elevated epidemic parotitis virus-specific IgM antibodies in the serum, (2) a 4-fold or higher elevation of epidemic parotitis virus-specific IgG antibody titers during the recovery or acute phases, and/or (3) isolated epidemic parotitis virus from body fluids, such as the saliva, urine, and cerebral spinal fluid.

### 2.3. Interest of Indicators and Definitions

We documented the earliest date of occurrence of relevant symptoms based on patient self-reporting as date of disease onset. Date of patient visiting hospital was the date when the patient firstly visited the hospital. Report date referred to the date when diseases were reported through the online disease surveillance/prevention and control system. Delayed hospital visiting was defined by the durations between the patient visiting date and the disease onset date over one day. Epidemic season of HFMD was defined from April to July and from November to December of each year [6], while the rest months of the year were considered as nonepidemic season. Similarly, the epidemic season for epidemic parotitis included April to July of each year and November to January of the following year, and the nonepidemic seasons included February to March and August to October of each year [6]. In addition, in the present study, the term “weekend” referred to both “natural weekend” and “nonnatural weekend” days, i.e., official festival or holiday break due to New Year's Day, Spring Festival, Qingming Festival, Labor Day, Dragon Boat Festival, Mid-Autumn Festival, and National Day in China. The holiday information was obtained from the “Notice on part of holiday arrangements” issued by the General Office of the State Council of the People's Republic of China each year. The day before the statutory holiday was defined as “Friday,” and the first return work day after each holiday was defined as “Monday.” The daily average numbers of cases reported on weekend referred to days of Saturday and Sunday defined above.

### 2.4. Statistical Analysis

The durations of patient delay in hospital visiting, i.e., 0-1 day, 2-3 days, 4–7 days, and >7 days, were described as frequencies and percentages, and the differences across factors were further compared using nonparametric tests. The odds ratio (OR) and its 95% confidence interval (CI) of these factors with the binary delayed hospital visiting were also estimated using multivariable logistic regression. The average number of daily disease onsets and reported cases on workdays and weekends were represented as the mean ± standard deviation, respectively.

A two-side *p* < 0.05 was regarded as statistical significance. All statistical analysis was performed using SPSS v.18.0 (IBM Corp., Armonk, NY, USA).

## 3. Results

### 3.1. Background Information of Included Patients

A total of 14,814 patients with HFMD were reported between January 1, 2014, and December 31, 2017, in Hanzhong, China. Among whom, 96.6% were clinically diagnosed cases, 96.3% of patients were children in or not in childcare center, and 88.0% were younger than five years of age (Table 1). Regarding epidemic parotitis, 4013 patients were recorded, among whom 99.2% were clinically diagnosed cases, 63.8% were students, and 52.7% were between 5 and 10 years of age (Table 1).

### 3.2. Durations of Delay between Disease Onset and Hospital Visiting

43.1% of patients with HFMD and 36.5% of patients with epidemic parotitis experienced delayed hospital visiting after disease onset. The percentages of durations of delay between disease onset and hospital visiting at 2-3, 4–7, and >7 days were 34.1%, 7.7%, and 1.3% for HFMD, respectively. The corresponding percentages for epidemic parotitis were 24.3%, 9.8%, and 2.3%, respectively. In addition, all patients were reported through the online disease surveillance system on the day when visiting the hospital.

#### 3.2.1. Factors Associated with Patient Delay in Hospital Visiting

The durations of delayed hospital visiting of both diseases differed significantly by years, ages, occupations, and epidemic seasons (Tables 2 and 3).

In the multivariable logistic models (Table 4), we found that the risks of delayed hospital visiting for HFMD in 2015, 2016, or 2017 were significantly higher as compared with that in 2014. The adjusted OR was 1.35 (95% CI: 1.23, 1.48), 1.16 (95% CI: 1.06, 1.27), and 1.16 (95% CI: 1.05, 1.27), respectively. Compared to children in childcare center, the risk of delayed hospital visiting increased by 41.2% (95% CI: 30.2%, 53.2%) and 32.2% (95% CI: 6.8%, 63.7%) among children who were not in childcare center and school students, respectively. We also found that the risk of occurring delayed hospital visiting in epidemic season was significantly lower than that in nonepidemic season with an adjusted OR of 0.78 (95% CI: 0.72, 0.85).

In terms of epidemic parotitis, the percentages of delayed hospital visiting increased significantly by years, ranging from 27.9% in 2014 to 40.1% in 2017. In addition, the risk of occurring delayed hospital visiting in epidemic season reduced by 15.1% (95% CI: 3.8%, 25.8%) when compared with nonepidemic season.

### 3.3. Distribution of Case Numbers and Reported Numbers within the Week

**Figure 1:**
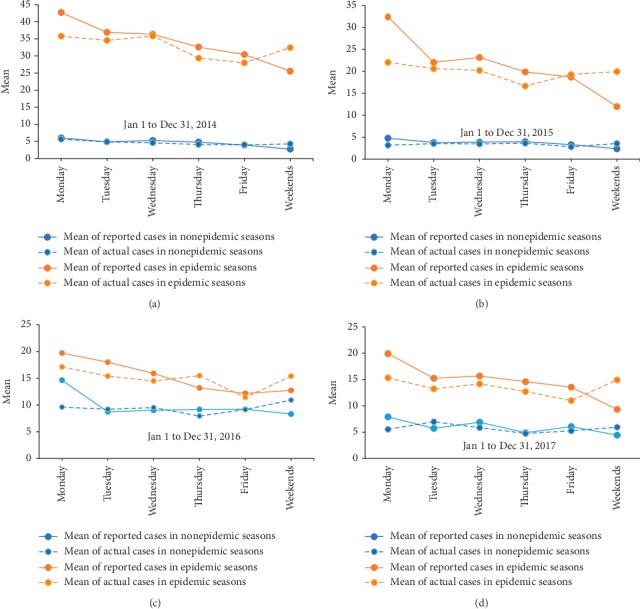
Distribution of weekly average actual cases and reported number of hand-foot-and-mouth disease cases between 2014 and 2017 in Hanzhong city, China.

**Figure 2:**
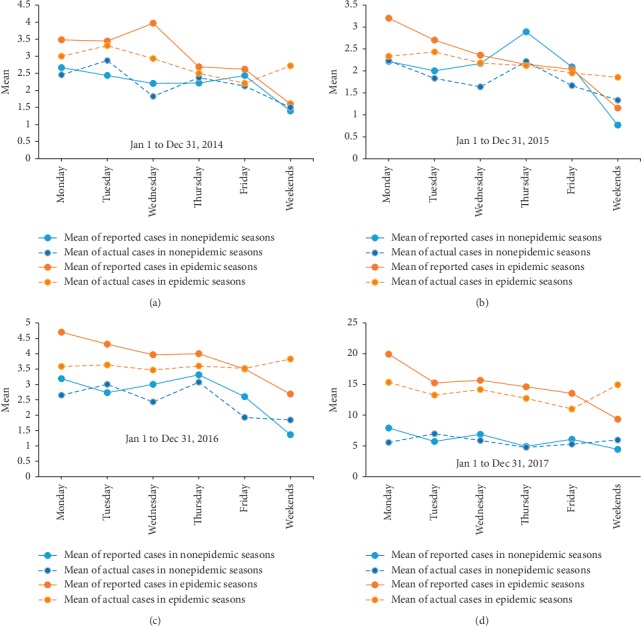
Distribution of weekly average actual cases and reported numbers of epidemic parotitis cases between 2014 and 2017 in Hanzhong city, China.

No obvious pattern of disease onset time was identified for HFMD or epidemic parotitis, and no weekend effect was detected. In contrast, the reported numbers of HFMD and epidemic parotitis cases both showed weekly patterns either in the epidemic or in the nonepidemic season, which decreased from Monday to the weekend with uniformly lower numbers on weekends than that on Monday (Figures 1 and 2, Supplementary Tables 1 and 2). Besides, the statistical tests to determine the difference of difference (DID) of reported and actual cases between weekend and weekday showed that the number of reported cases on weekends was uniformly and significantly lower than that of actual cases during weekdays. The mean (SD) of DID was 0.79 (3.27) for epidemic parotitis and 1.44 (3.31) for hand-foot-and-mouth disease on weekends, with corresponding *p* values of <0.001 and<0.001, respectively.

## 4. Discussion

### 4.1. Main Findings

We found that 88.0% of HFMD cases were children younger than 5 years of age between 2014 and 2017. In contrast, the majority of the patients with epidemic parotitis were school students (63.8%), with children aged 5–10 years accounting for 52.7%. We also found that 43.1% of patients with HFMD and 36.5% of patients with epidemic parotitis had delays in hospital visiting in nonepidemic seasons, which was slightly reduced, but similar issue still identified in epidemic seasons. Majority of the durations of delaying hospital visiting lied within two to three days after disease onset. Besides, the risk of delays in hospital visiting for epidemic parotitis showed an increasing trend with the years. We did not identify the weekend effect on the time of disease onset for both the diseases above. However, we found an obvious weekend effect on the time of online reporting with the significantly lowest number of cases reported over the weekend and the highest reported on Monday, which resulted in a phenomenon of Monday jump in case reports.

### 4.2. Interpretations of the Findings

Both the HFMD and epidemic parotitis were infectious diseases and included in Chinese statutory management. We found that the absolute number of mumps cases consistently increased along with years from 2014 to 2017, but we were not able to report the incidence rate. Su and the coauthors reported a steady incidence of mumps in China between 2004 and 2013 [19], surrounding around the rate of 22/100,000, and no national report after 2013 was identified. In the present study, 52.7% and 34.3% of the mumps cases were identified as school students and aged 5 to 10 and above 10 years old, respectively, which was similar to the national report [19, 20], suggesting the sound and reliable quality of data used. Of note, this epidemiological characteristic of age peak is different from that in other developed countries [21–24], where two-dose mumps vaccine has been routinely applied among children, which is usually outbreak among young adults. However, in China, the coverage of the second dose was relatively low, only 78.8% reported in some studies [25]. Besides, Liu and colleagues reported that waning immunity to mumps among infants who received one-dose vaccine during 18–24 months of age occurred with increasing age [26], resulting in the higher incidence rate in primary schools identified in the present study. These results suggest that improving the coverage of the second dose of vaccine should be prioritized to prevent mumps epidemics in China. Besides, the third dose may be also necessary given the reemergence among adults in developed countries [21, 23].

Due to no routine vaccine for preventing HFMD [27], much more HFMD cases than mumps were reported in the present study. The incidence of HMFD showed an increasing tendency in China from 2008 to 2017 [9], but no corresponding increment of absolute case numbers was found in the present study. Again, we were not able to report the incidence rate which warranted further study and explanations. As was reported in the national report [9], the HFMD patients targeted on young infants aged less than 4 years old, accounting for 88% of the total cases. This population characteristic also explained that majority of the cases were found among children not in childcare center. In China, the childcare center usually accepts the infants aged 3 years above. Consequently, the development of strategies and programs for preventing and controlling such diseases should target on parents and caregivers.

To our knowledge, this is the first study to report that a fairly high percentage of patients had delays in hospital visiting for HFMD and mumps in China, as high as 43.1% for HFMD and 36.5% for epidemic parotitis, respectively. This delay in hospital visiting fails to make the timely notification to a local Center for Disease Control and Prevention, with extremely high probabilities of resulting in rapidly spreading within facilities and local outbreaks, even though majority of the cases had delayed durations of 2 to 3 days. Besides, the delay in hospital visiting and care at home might increase the risk of developing severe symptoms [28]. A similar situation was also reported in other infectious diseases like tuberculosis [29, 30]. In addition, we found that patients occurred in nonepidemic seasons were more likely to experience the delay in hospital visiting, which implied that caregivers were less alert and might ignore the mild symptoms at the early stage of these diseases during nonepidemic seasons. We also found that children taken care at home were more likely to have delays in hospital visiting as compared with those in childcare center, where the morning and noon infectious disease inspection system was implemented and the staff were trained for screening and identifying the early signs, which suggested the necessities of improving related knowledge among caregivers. In the present study, it was also worthy to note that the risks of delay in hospital visiting for epidemic parotitis increased by years, which might be due to the decreasing of cases with typical clinical manifestations in recent years, especially among vaccinated populations [31]. In contrast, the incidence of HFMD kept at a much higher level that caused high concerns of all parties and strengthened the knowledge on prevention and treatment. This hypothesis was also supported by the finding that the risk of occurring delays in hospital visiting for HFMD kept steady in recent years. Taken together, given the fact that majority of cases have mild symptoms and that caregivers lack necessary knowledge on signs and symptoms at the early stage of these diseases, these results suggest the emphasis on community surveillance, prevention, and control especially in nonepidemic seasons [32]. Based on a community intervention trial, Guo and the coauthors reported that intensive education on handwashing for parents and caregivers of children aged 6 to 40 months in villages effectively reduced the incidence of HFMD [33], suggesting the feasibility and practicality of the community implementation for preventing these diseases and/or reducing the risk of delay in hospital visiting after disease onset.

In addition, we found that the online reports but not disease onset of cases showed a significant weekend effect for both HFMD and epidemic parotitis. Weekend effect is well noted in the context of hospital admissions of noncommunicable diseases such as emergency surgery and stroke, where patient has worse outcomes on weekend hospital admissions as compared with those on weekdays [34]. Nevertheless, the weekend effect is less studied in the infectious disease area, with a slightly different meaning in the present study, which indicates that the number of cases not reported on weekends is significantly higher than that on weekdays. As discussed in the delaying of hospital visiting above, these cases miss to report may lead to the local outbreak and severe symptoms, which warrants notice of related organizations. In terms of causes of weekend effect, two primary domains were investigated and discussed in the literature studies [35], patients themselves (parents and caregivers in the context of our study) and health system. Amirov and colleagues also reported that Mondays and Fridays had the highest occurrence of outbreak reporting in healthcare facilities from Toronto, Canada [36], and the authors concluded the health staff differences between weekend and weekdays as one of the causes. Although we were not able to determine the causes in the present study, health system might not be the factor in the causal link because we found that all cases were reported through the online disease surveillance system on the same day when patient visiting the hospital. Actually, to reduce deaths associated with the weekend effect, National Health Service in the UK implemented four priority clinical standards for a balanced seven-day hospital care. However, Meacock and Sutton did not find any significant changes in weekend mortality after three years' adoption of these clinical standards [37]. Besides, based on the data of unselected emergency admissions to four large Oxford hospitals between January 2006 and December 2014, Walker and colleagues adjusted for 15 common hematology and biochemistry test results not considered in other studies and found that these routine test results of patients substantially reduced excess mortality associated with emergency admission at weekends and public holidays [38]. Our finding that patients had delaying presenting to hospital visiting also indirectly supported this assumption. That said, a similar seven-day hospitalist program implemented in Taiwan showed potential for equaling the patient outcomes between weekday and weekend general medicine admissions, with a fairly smaller sample size of 861 [39]. Notwithstanding, from the perspective of health economy, Meacock and colleagues found that these programs were not cost-effective, i.e., the planned cost of implementing 7-day services substantially exceeding the maximum amount that the National Health Service in the UK expected to eradicate the weekend effect [40]. Arguably, given the current evidence, patient-level differences rather than reduced health system and/or staffing levels play the key role in the context of weekend effect and are worthy to further examine the underlying mechanisms. For example, children playing activities not at home may increase the chances of contacts and being infected over the weekends, which consequently leads to the disease onset and more cases at the beginning of the week, resulting in the data artefact, i.e., fewer cases reported on weekends.

### 4.3. Implications for Surveillance, Prevention, and Control of Infectious Diseases

Recently in China, programs on public health education are continuously implemented and strengthened through various methods. However, it is difficult and challenging for the public and caregivers to know and master every piece of health knowledge due to the complicated and professional features of health knowledge. Consequently, we encouraged that the stratified and targeted education on infectious diseases that were common in children in corresponding ages should be developed and implemented in China, especially focusing on nonepidemic seasons and infectious diseases with low incidence rate. These strategies would effectively help caregivers master the corresponding prevention and control knowledge of infectious diseases such as early signs and thus improve the behavior of timely seeking medical service. Besides, these strategies also could help to reduce the detrimental effects of the weekend effects and ensure that the reported case numbers objectively indicated the actual status of disease incidence. In addition, when analyzing the surveillance data, the “weekend effect” should be considered using method like 7-day moving summation [17].

## 5. Strengths and Limitations

The surveillance data used in this study ensured the data quality and convincing results. However, this study has some limitations. First, we focused on the timing of the surveillance system but were unable to evaluate the accuracy and completeness of the surveillance data. As a result, the quality assessment of the entire surveillance system was not sufficiently thorough. Second, the data source did not allow us to examine the causes and/or consequences of the weekend effect that warrants further studies. Finally, we were unable to determine whether the disease was on outbreak that also may affect the quality assessment of the surveillance system.

## 6. Conclusions

The reported cases of HFMD and epidemic parotitis had an obvious weekend effect, with an increasing tendency of cases delaying in hospital visiting over the recent years in Hanzhong, China. Parents and caregivers rather than health systems should be primarily targeted for the prevention and control of infectious diseases and their local outbreaks such as community-based education on the second-dose vaccination of mumps and/or hand hygiene.

## Figures and Tables

**Table 1:** Background information of patients with hand-foot-and-mouth disease or epidemic parotitis.

Items	Hand-foot-and-mouth disease (*n* (%))	Epidemic parotitis (*n* (%))
Year		
2014	5067 (34.2)	774 (19.3)
2015	3183 (21.5)	548 (13.7)
2016	3650 (24.6)	1020 (25.7)
2017	2914 (19.7)	1671 (41.6)

Gender		
Male	8696 (58.7)	2266 (56.5)
Female	6118 (41.3)	1747 (43.5)

Classification of disease		
Clinically diagnosed cases	14,311 (96.6)	3981 (99.2)
Confirmed cases	503 (3.4)	32 (0.8)

Occupation		
Children in childcare	4420 (29.8)	664 (16.5)
Children not in childcare	9706 (65.5)	255 (6.4)
Students	632 (4.3)	2561 (63.8)
Peasants	29 (0.2)	407 (10.1)
Others	27 (0.2)	126 (3.1)

Age		
<1 year	1592 (10.7)	8 (0.2)
1–4 years	11,457 (77.3)	512 (12.8)
5–10 years	1585 (10.7)	2115 (52.7)
>10 years	180 (1.2)	1378 (34.3)

**Table 2:** Comparisons of durations of delay in patient hospital visiting for hand-foot-and-mouth disease across different factors.

Factors	Durations of delay in patient hospital visiting (days)				Statistics	*p* values
	0-1	2-3	4–7	>7		
Year					90.532	<0.001
2014	3077 (60.7)	1569 (31.0)	361 (7.1)	60 (1.2)		
2015	1679 (52.7)	1210 (38.0)	274 (8.6)	20 (0.6)		
2016	2022 (55.4)	1242 (34.0)	323 (8.8)	63 (1.7)		
2017	1649 (56.6)	1032 (35.4)	188 (6.5)	45 (1.5)		

Gender					3.458	0.326
Male	4968 (57.1)	2974 (34.2)	643 (7.4)	111 (1.3)		
Female	3459 (56.5)	2079 (34.0)	503 (8.2)	77 (1.3)		

Classification of disease					300.086	<0.001
Clinically diagnosed cases	8157 (57.0)	4897 (34.2)	1118 (7.8)	139 (1.0)		
Confirmed cases	270 (53.7)	156 (31.0)	28 (5.6)	49 (9.7)		

Occupation					133.591	<0.001
Children in childcare	2799 (63.3)	1335 (30.2)	252 (5.7)	34 (0.8)		
Children not in childcare	5269 (54.3)	3459 (35.6)	831 (8.6)	147 (1.5)		
Students	340 (53.8)	231 (36.6)	54 (8.5)	7 (1.1)		
Peasants and others	19 (33.9)	28 (50.0)	9 (16.1)	0 (0.0)		

Age					45.480	<0.001
<1 year	857 (53.8)	586 (36.8)	126 (7.9)	23 (1.4)		
1–4 years	6533 (57.0)	3890 (34.0)	883 (7.7)	151 (1.3)		
5–10 years	967 (61.0)	488 (30.8)	117 (7.4)	13 (0.8)		
>10 years	70 (38.9)	89 (49.4)	20 (11.1)	1 (0.6)		

Epidemic season					56.490	<0.001
Yes	6821 (58.3)	3913 (33.4)	842 (7.2)	132 (1.1)		
No	1606 (51.7)	1140 (36.7)	304 (9.8)	56 (1.8)		

**Table 3:** Comparisons of durations of delay in patient hospital visiting for epidemic parotitis across different factors.

Factors	Durations of delay in patient hospital visiting (days)				Statistics	*p* values
	0–1	2-3	4–7	>7		
Year					48.823	<0.001
2014	558 (72.1)	159 (20.5)	44 (5.7)	13 (1.7)		
2015	356 (65.0)	142 (25.9)	42 (7.7)	8 (1.5)		
2016	635 (62.3)	252 (24.7)	104 (10.2)	29 (2.8)		
2017	1001 (59.9)	424 (25.4)	202 (12.1)	44 (2.6)		

Gender					1.749	0.626
Male	1439 (63.5)	541 (23.9)	229 (10.1)	57 (2.5)		
Female	1111 (63.6)	436 (25.0)	163 (9.3)	37 (2.1)		

Classification of disease					3.089	0.378
Clinically diagnosed cases	2533 (63.6)	968 (24.3)	388 (9.7)	92 (2.3)		
Confirmed cases	17 (53.1)	9 (28.1)	4 (12.5)	2 (6.2)		

Occupation					22.149	0.008
Children in childcare	455 (68.5)	150 (22.6)	50 (7.5)	9 (1.4)		
Children not in childcare	165 (64.7)	65 (25.5)	19 (7.5)	6 (2.4)		
Students	1611 (62.9)	623 (24.3)	270 (10.5)	57 (2.2)		
Peasants and others	319 (59.8)	139 (26.1)	53 (9.9)	22 (4.1)		

Age					22.431	0.008
<1 year	4 (50.0)	2 (25.0)	2 (25.0)	0 (0.0)		
1–4 years	344 (66.9)	124 (24.2)	34 (6.6)	10 (2.0)		
5–10 years	1377 (65.1)	494 (23.4)	203 (9.6)	41 (1.9)		
>10 years	825 (59.9)	357 (25.9)	153 (11.1)	43 (3.1)		

Epidemic season					8.227	0.042
Yes	1663 (64.8)	616 (24.0)	234 (9.1)	52 (2.0)		
No	887 (61.3)	361 (24.9)	158 (10.9)	42 (2.9)		

**Table 4:** Multivariable logistic regressions on associations of influencing factors with patients delayed in hospital visiting for hand-foot-and-mouth disease (HFMD) and epidemic parotitis.

Disease	Factors	*n* (%)	*B*	SE	Wals	Sig.	OR	95% CI for the OR	
								Lower limit	Upper limit
HFMD	*Year*								
	2014	1990 (39.3)					Ref		
	2015	1504 (47.3)	0.298	0.046	41.449	<0.001	1.347	1.230	1.475
	2016	1628 (44.6)	0.147	0.045	10.681	0.001	1.158	1.061	1.265
	2017	1265 (43.4)	0.145	0.048	9.254	0.002	1.156	1.053	1.270
	*Gender*								
	Male	3728 (42.9)					Ref		
	Female	2659 (43.5)	0.025	0.034	0.525	0.469	1.025	0.959	1.096
	*Classification of disease*								
	Clinically diagnosed cases	6154 (43.0)	−0.171	0.092	3.445	0.063	0.843	0.704	1.010
	Confirmed cases	233 (46.3)					Ref		
	*Occupation*								
	Children in childcare	1621 (36.7)					Ref		
	Children not in childcare	4437 (45.7)	0.345	0.042	69.044	<0.001	1.412	1.302	1.532
	Students	292 (46.2)	0.279	0.109	6.595	0.010	1.322	1.068	1.637
	Peasants and others	37 (66.1)	0.513	0.354	2.099	0.147	1.671	0.834	3.346
	*Age*								
	<1 year	735 (46.2)					Ref		
	1–4 years	4924 (43.0)	−0.009	0.055	0.035	0.876	0.991	0.890	1.105
	5–10 years	618 (39.0)	0.001	0.089	0.001	0.995	1.001	0.841	1.191
	>10 years	110 (61.1)	0.640	0.222	8.324	0.004	1.897	1.228	2.931
	*Epidemic season*								
	Yes	4887 (41.7)	−0.246	0.042	35.122	<0.001	0.782	0.721	0.848
	No	1500 (48.3)					Ref		
Epidemic parotitis	*Year*								
	2014	216 (27.9)					Ref		
	2015	192 (35.0)	0.351	0.121	8.423	0.004	1.420	1.121	1.800
	2016	385 (37.7)	0.483	0.104	21.458	<0.001	1.621	1.322	1.989
	2017	670 (40.1)	0.556	0.096	33.708	<0.001	1.743	1.445	2.102
	*Gender*								
	Male	827 (36.5)					Ref		
	Female	636 (36.4)	−0.033	0.068	0.234	0.628	0.968	0.848	1.105
	*Classification of disease*								
	Clinically diagnosed cases	1448 (36.4)	−0.392	0.359	1.193	0.275	0.676	0.334	1.365
	Confirmed cases	15 (46.9)					Ref		
	*Occupation*								
	Children in childcare	209 (31.5)					Ref		
	Children not in childcare	90 (35.3)	0.189	0.172	1.209	0.272	1.208	0.863	1.691
	Students	950 (37.1)	0.112	0.121	0.856	0.355	1.118	0.883	1.416
	Peasants and others	214 (40.2)	0.166	0.166	0.479	0.489	1.122	0.810	1.555
	*Age*								
	<1 year	4 (50.0)					Ref		
	1–4 years	168 (32.8)	−0.678	0.724	0.878	0.349	0.508	0.123	2.097
	5–10 years	738 (34.9)	−0.648	0.736	0.775	0.379	0.523	0.124	2.213
	>10 years	553 (40.1)	−0.426	0.741	0.331	0.565	0.653	0.153	2.790
	*Epidemic season*								
	Yes	902 (35.2)	−0.163	0.069	5.637	0.018	0.849	0.742	0.972
	No	561 (38.7)					Ref		

## Data Availability

Data on HFMD and epidemic parotitis were obtained from the “Infectious disease report information management system” subsystem of the “China disease prevention and control information system.”

## Abbreviations

HFMD: Hand-foot-and-mouth disease.

## Supplementary Materials

Supplementary Materials Supplementary Table 1: weekly average of actual cases and reported numbers of epidemic parotitis between 2014 and 2017 in Hanzhong city, China. Supplementary Table 2: weekly average of actual cases and reported numbers of hand-foot-and-mouth disease between 2014 and 2017 in Hanzhong city, China.
