# Supplementary material for: Patient Delay in Hospital Visiting and the Weekend Effect of Surveillance Report on Hand-Foot-and-Mouth Disease and Epidemic Parotitis in Hanzhong City, China
Source: Can J Infect Dis Med Microbiol. 2020 May 6;2020:7081219. doi: 10.1155/2020/7081219 (PMC7225908; doi:10.1155/2020/7081219)
Supplement: Supplementary Materials — Supplementary Table 1: weekly average of actual cases and reported numbers of epidemic parotitis between 2014 and 2017 in Hanzhong city, China. Supplementary Table 2: weekly average of actual cases and reported numbers of hand-foot-and-mouth disease between 2014 and 2017 in Hanzhong city, China. [file 7081219.f1.docx]

Supplementary table 1 Weekly average of actual cases and reported numbers of epidemic parotitis between 2014 and 2017 in Hanzhong city, China

| Year | Epidemic season | Week | Report cases | | | Actual cases | | |
| --- | --- | --- | --- | --- | --- | --- | --- | --- |
|  |  |  | Total | Mean | SD | Total | Mean | SD |
| 2014 | No | Monday | 32 | 2.67 | 1.72 | 27 | 2.45 | 1.69 |
| 2014 | No | Tuesday | 39 | 2.44 | 1.36 | 46 | 2.88 | 1.45 |
| 2014 | No | Wednesday | 33 | 2.20 | 1.57 | 31 | 1.82 | 0.88 |
| 2014 | No | Thursday | 31 | 2.21 | 0.89 | 38 | 2.38 | 1.26 |
| 2014 | No | Friday | 39 | 2.44 | 0.81 | 34 | 2.13 | 1.09 |
| 2014 | No | Weekends | 27 | 1.39 | 0.81 | 29 | 1.50 | 0.80 |
| 2014 | Yes | Monday | 94 | 3.48 | 2.34 | 72 | 3.00 | 1.89 |
| 2014 | Yes | Tuesday | 93 | 3.44 | 3.68 | 96 | 3.31 | 2.99 |
| 2014 | Yes | Wednesday | 115 | 3.97 | 3.83 | 88 | 2.93 | 2.30 |
| 2014 | Yes | Thursday | 78 | 2.69 | 2.24 | 60 | 2.50 | 1.50 |
| 2014 | Yes | Friday | 76 | 2.62 | 1.45 | 53 | 2.21 | 1.41 |
| 2014 | Yes | Weekends | 47 | 1.60 | 1.30 | 87 | 2.72 | 1.68 |
| 2015 | No | Monday | 31 | 2.21 | 1.19 | 29 | 2.23 | 1.48 |
| 2015 | No | Tuesday | 28 | 2.00 | 1.47 | 22 | 1.83 | 1.19 |
| 2015 | No | Wednesday | 26 | 2.17 | 1.47 | 18 | 1.64 | 0.67 |
| 2015 | No | Thursday | 26 | 2.89 | 1.54 | 31 | 2.21 | 1.05 |
| 2015 | No | Friday | 23 | 2.09 | 1.14 | 20 | 1.67 | 0.89 |
| 2015 | No | Weekends | 12 | 0.77 | 0.32 | 20 | 1.33 | 0.72 |
| 2015 | Yes | Monday | 80 | 3.20 | 1.85 | 63 | 2.33 | 1.30 |
| 2015 | Yes | Tuesday | 81 | 2.70 | 1.29 | 73 | 2.43 | 1.48 |
| 2015 | Yes | Wednesday | 59 | 2.36 | 1.50 | 48 | 2.18 | 1.33 |
| 2015 | Yes | Thursday | 58 | 2.15 | 1.26 | 53 | 2.12 | 1.36 |
| 2015 | Yes | Friday | 53 | 2.04 | 1.28 | 43 | 1.95 | 1.13 |
| 2015 | Yes | Weekends | 34 | 1.16 | 0.58 | 58 | 1.85 | 0.91 |
| 2016 | No | Monday | 51 | 3.19 | 1.83 | 45 | 2.65 | 1.32 |
| 2016 | No | Tuesday | 41 | 2.73 | 1.28 | 48 | 3.00 | 2.03 |
| 2016 | No | Wednesday | 36 | 3.00 | 2.17 | 34 | 2.43 | 2.10 |
| 2016 | No | Thursday | 53 | 3.31 | 2.15 | 43 | 3.07 | 2.30 |
| 2016 | No | Friday | 39 | 2.60 | 1.55 | 25 | 1.92 | 0.95 |
| 2016 | No | Weekends | 25 | 1.36 | 0.64 | 35 | 1.84 | 0.75 |
| 2016 | Yes | Monday | 141 | 4.70 | 2.39 | 104 | 3.59 | 2.18 |
| 2016 | Yes | Tuesday | 125 | 4.31 | 2.78 | 109 | 3.63 | 3.16 |
| 2016 | Yes | Wednesday | 123 | 3.97 | 2.50 | 104 | 3.47 | 2.62 |
| 2016 | Yes | Thursday | 120 | 4.00 | 2.60 | 108 | 3.60 | 2.14 |
| 2016 | Yes | Friday | 84 | 3.50 | 2.34 | 88 | 3.52 | 2.63 |
| 2016 | Yes | Weekends | 81 | 2.68 | 1.47 | 123 | 3.83 | 1.80 |
| 2017 | No | Monday | 74 | 3.89 | 2.08 | 65 | 3.61 | 2.38 |
| 2017 | No | Tuesday | 81 | 4.50 | 2.38 | 76 | 4.22 | 3.12 |
| 2017 | No | Wednesday | 69 | 3.63 | 3.24 | 47 | 2.76 | 1.71 |
| 2017 | No | Thursday | 62 | 3.65 | 2.18 | 50 | 3.13 | 1.50 |
| 2017 | No | Friday | 44 | 2.59 | 1.33 | 63 | 3.71 | 1.93 |
| 2017 | No | Weekends | 41 | 2.03 | 1.07 | 60 | 3.00 | 1.49 |
| 2017 | Yes | Monday | 195 | 7.22 | 3.25 | 177 | 6.56 | 2.87 |
| 2017 | Yes | Tuesday | 211 | 7.54 | 2.90 | 166 | 5.93 | 2.62 |
| 2017 | Yes | Wednesday | 187 | 6.03 | 3.06 | 177 | 5.71 | 2.60 |
| 2017 | Yes | Thursday | 194 | 6.26 | 3.39 | 184 | 5.94 | 3.70 |
| 2017 | Yes | Friday | 195 | 6.50 | 2.89 | 163 | 5.26 | 3.32 |
| 2017 | Yes | Weekends | 141 | 4.41 | 2.64 | 194 | 6.05 | 2.70 |

Supplementary table 2 Weekly average of actual cases and reported numbers of hand-foot-and-mouth disease between 2014 and 2017 in Hanzhong city, China

| Year | Epidemic season | Week | Report cases | | | Actual cases | | |
| --- | --- | --- | --- | --- | --- | --- | --- | --- |
|  |  |  | Total | Mean | SD | Total | Mean | SD |
| 2014 | No | Monday | 180 | 6.00 | 6.71 | 157 | 5.61 | 4.76 |
| 2014 | No | Tuesday | 146 | 4.87 | 5.27 | 126 | 4.85 | 3.90 |
| 2014 | No | Wednesday | 120 | 5.22 | 3.97 | 123 | 4.56 | 3.07 |
| 2014 | No | Thursday | 143 | 4.77 | 4.92 | 114 | 4.07 | 4.44 |
| 2014 | No | Friday | 113 | 3.90 | 2.29 | 108 | 4.00 | 3.57 |
| 2014 | No | Weekends | 84 | 2.71 | 2.44 | 132 | 4.24 | 4.29 |
| 2014 | Yes | Monday | 769 | 42.72 | 22.67 | 644 | 35.78 | 20.73 |
| 2014 | Yes | Tuesday | 702 | 36.95 | 18.92 | 657 | 34.58 | 18.39 |
| 2014 | Yes | Wednesday | 691 | 36.37 | 16.55 | 681 | 35.84 | 16.91 |
| 2014 | Yes | Thursday | 587 | 32.61 | 15.35 | 529 | 29.39 | 16.15 |
| 2014 | Yes | Friday | 548 | 30.44 | 17.05 | 504 | 28.00 | 12.31 |
| 2014 | Yes | Weekends | 486 | 25.55 | 17.09 | 616 | 32.42 | 18.58 |
| 2015 | No | Monday | 129 | 4.78 | 3.68 | 83 | 3.19 | 2.17 |
| 2015 | No | Tuesday | 99 | 3.81 | 2.42 | 85 | 3.54 | 2.75 |
| 2015 | No | Wednesday | 97 | 3.88 | 2.15 | 77 | 3.50 | 2.09 |
| 2015 | No | Thursday | 92 | 4.00 | 2.63 | 87 | 3.63 | 2.57 |
| 2015 | No | Friday | 80 | 3.33 | 2.16 | 68 | 2.83 | 2.06 |
| 2015 | No | Weekends | 69 | 2.38 | 1.88 | 112 | 3.60 | 2.58 |
| 2015 | Yes | Monday | 551 | 32.41 | 19.01 | 375 | 22.06 | 12.29 |
| 2015 | Yes | Tuesday | 419 | 22.05 | 9.27 | 392 | 20.63 | 11.32 |
| 2015 | Yes | Wednesday | 440 | 23.16 | 12.86 | 384 | 20.21 | 9.86 |
| 2015 | Yes | Thursday | 378 | 19.89 | 10.98 | 317 | 16.68 | 9.16 |
| 2015 | Yes | Friday | 318 | 18.71 | 7.99 | 348 | 19.33 | 11.78 |
| 2015 | Yes | Weekends | 228 | 12.00 | 6.72 | 379 | 19.95 | 10.17 |
| 2016 | No | Monday | 380 | 14.62 | 12.74 | 259 | 9.59 | 7.75 |
| 2016 | No | Tuesday | 253 | 8.72 | 7.97 | 239 | 9.19 | 7.40 |
| 2016 | No | Wednesday | 243 | 9.00 | 5.88 | 257 | 9.52 | 6.71 |
| 2016 | No | Thursday | 238 | 9.15 | 7.43 | 214 | 7.93 | 6.67 |
| 2016 | No | Friday | 239 | 9.19 | 7.33 | 210 | 9.13 | 6.48 |
| 2016 | No | Weekends | 233 | 8.30 | 8.77 | 317 | 10.91 | 10.76 |
| 2016 | Yes | Monday | 335 | 19.71 | 7.61 | 291 | 17.12 | 6.17 |
| 2016 | Yes | Tuesday | 324 | 18.00 | 7.64 | 277 | 15.39 | 5.67 |
| 2016 | Yes | Wednesday | 286 | 15.89 | 7.97 | 261 | 14.50 | 8.96 |
| 2016 | Yes | Thursday | 224 | 13.18 | 5.43 | 263 | 15.47 | 5.49 |
| 2016 | Yes | Friday | 207 | 12.18 | 5.89 | 194 | 11.41 | 6.15 |
| 2016 | Yes | Weekends | 229 | 12.72 | 6.70 | 277 | 15.39 | 5.99 |
| 2017 | No | Monday | 245 | 7.90 | 6.36 | 172 | 5.55 | 5.33 |
| 2017 | No | Tuesday | 183 | 5.72 | 4.75 | 195 | 6.96 | 5.63 |
| 2017 | No | Wednesday | 186 | 6.89 | 5.87 | 170 | 5.86 | 4.67 |
| 2017 | No | Thursday | 157 | 4.91 | 3.63 | 152 | 4.75 | 3.78 |
| 2017 | No | Friday | 188 | 6.06 | 4.65 | 163 | 5.26 | 3.72 |
| 2017 | No | Weekends | 146 | 4.42 | 4.00 | 203 | 5.96 | 5.68 |
| 2017 | Yes | Monday | 299 | 19.93 | 7.80 | 230 | 15.33 | 6.78 |
| 2017 | Yes | Tuesday | 244 | 15.25 | 5.52 | 212 | 13.25 | 7.42 |
| 2017 | Yes | Wednesday | 282 | 15.67 | 7.19 | 255 | 14.17 | 5.65 |
| 2017 | Yes | Thursday | 263 | 14.61 | 6.97 | 229 | 12.72 | 5.49 |
| 2017 | Yes | Friday | 244 | 13.56 | 6.23 | 198 | 11.00 | 5.59 |
| 2017 | Yes | Weekends | 168 | 9.33 | 5.35 | 269 | 14.94 | 6.54 |
